# BABA and *Phytophthora nicotianae* Induce Resistance to *Phytophthora capsici* in Chile Pepper (*Capsicum annuum*)

**DOI:** 10.1371/journal.pone.0128327

**Published:** 2015-05-28

**Authors:** Rio A. Stamler, Omar Holguin, Barry Dungan, Tanner Schaub, Soumaila Sanogo, Natalie Goldberg, Jennifer J. Randall

**Affiliations:** 1 The Department of Entomology, Plant Pathology, and Weed Science, New Mexico State University, Las Cruces, New Mexico, United States of America; 2 The Department of Plant and Environmental Sciences, New Mexico State University, Las Cruces, New Mexico, United States of America; 3 The Department of Extension Plant Sciences, New Mexico State University, Las Cruces, New Mexico, United States of America; 4 Chemical Analysis and Instrumentation Laboratory, New Mexico State University, Las Cruces, New Mexico, United States of America; University of Nebraska-Lincoln, UNITED STATES

## Abstract

Induced resistance in plants is a systemic response to certain microorganisms or chemicals that enhances basal defense responses during subsequent plant infection by pathogens. Inoculation of chile pepper with zoospores of non-host *Phytophthora nicotianae* or the chemical elicitor beta-aminobutyric acid (BABA) significantly inhibited foliar blight caused by *Phytophthora capsici*. Tissue extract analyses by GC/MS identified conserved change in certain metabolite concentrations following *P*. *nicotianae* or BABA treatment. Induced chile pepper plants had reduced concentrations of sucrose and TCA cycle intermediates and increased concentrations of specific hexose-phosphates, hexose-disaccharides and amino acids. Galactose, which increased significantly in induced chile pepper plants, was shown to inhibit growth of *P*. *capsici* in a plate assay.

## Introduction

*Phytophthora capsici* is an economically significant oomycete plant pathogen that impacts production of several crops worldwide, especially solanaceous and cucurbitaceous vegetable crops. With adequate moisture, this pathogen causes Phytophthora blight, which affects all aboveground and belowground parts of susceptible hosts [1]. Under conducive environmental conditions, *P*. *capsici* can cause up to 50% yield loss on chile pepper (*Capsicum annuum*), an economically important crop in the Southwest United States [2].

Hemibiotrophic in nature, *P*. *capsici* establishes infection through haustoria-like structures and intercellular hyphael growth and initiates host cell-death within 48 h of successful colonization [3]. Current management of this pathogen, including the use of fungicides and tolerant cultivars, is limited in terms of reducing Phytophthora blight due to the significant amount of genetic diversity in populations of *P*. *capsici*, and the ability of the pathogen to rapidly produce large numbers of propagules on infected plants [1]. Exploration and identification of new approaches, such as induced resistance, are continually needed to reduce the impact of *P*. *capsici* on various crops.

Induced resistance or systemic acquired resistance is a well-characterized response in *C*. *annuum* to non-host pathogenic microorganisms. Inoculation of *C*. *annuum* with an avirulent strain of *X*. *campestris* induced the systemic expression of pathogenesis related (PR) gene transcripts, microoxidative burst, and induction of ion-leakage and callose deposition in non-inoculated leaves [4]. Inoculation of *C*. *annuum* with the non-host pathogen *Fusarium oxysporum*, significantly inhibited subsequent infection by *P*. *capsici*, *Verticillium dahliae*, and *Botrytis cinerea*, and was associated with increased chitinase activity and cell-wall bound phenolics [5]. The chemical elicitor β-amino-butyric acid (BABA) is a non-protein amino acid that induces a plant systemic defense response against subsequent infection by multiple plant pathogens [6]. *Arabidopsis thaliana* mutant line analysis and the use of chemical inhibitors demonstrated that BABA-induced resistance is based on abscisic acid dependent priming for callose production and is independent of jasmonic acid (JA), salicylic acid (SA), or ethylene (ET) production [7–8].

In Europe, *P*. *nicotianae* is a known pathogen on *C*. *annuum* where it causes root- and collar-rot in field-grown plants [9]. *Phytophthora nicotianae* isolates recovered from diseased *C*. *annuum* plants in northern Spain were all virulent on selected *C*. *annuum* plants while a reference *P*. *nicotianae* isolate was not virulent on any varieties tested, indicating differences in host range among *P*. *nicotianae* isolates [10]. To our knowledge, *P*. *nicotianae* has not been reported to cause disease in *C*. *annuum* in North America. *P*. *nicotianae* was first reported in New Mexico on onion and tomato in 2011 [11–12]. Given the importance of chile pepper production to New Mexico, *P*. *nicotianae* isolates from onion and tomato were tested for pathogenicity on *C*. *annuum* where they failed to produce disease symptoms at any inoculum level, indicating a non-host interaction.

Pathogenicity assays determine that pre-inoculation with *P*. *nicotianae* or treatment with BABA induced a cultivar independent systemic response in *C*. *annuum*, which inhibited foliar infection by *P*. *capsici*. Fluorescence microscopy and the use of H_2_O_2_ substrates identifies a potentiated induction of cell wall reinforcements and the rapid production of oxidizing compounds that leads to a hypersensitive response. Additionally, significant change in metabolite concentrations in response to BABA treatment and inoculation with non-host *P*. *nicotianae* (including changes in hexose sugars, aromatic amino acids, and glycerol 3-phosphate) are identified by GC/MS metabolite analysis. In particular, galactose concentration, which increased for induced *C*. *annuum* plants, was shown to inhibit mycelial growth of *P*. *capsici* in an *in-vitro* plate assay.

## Results

### *Phytophthora nicotianae* isolate NMT1 is not pathogenic on *C*. *annuum*

**Fig 1:**
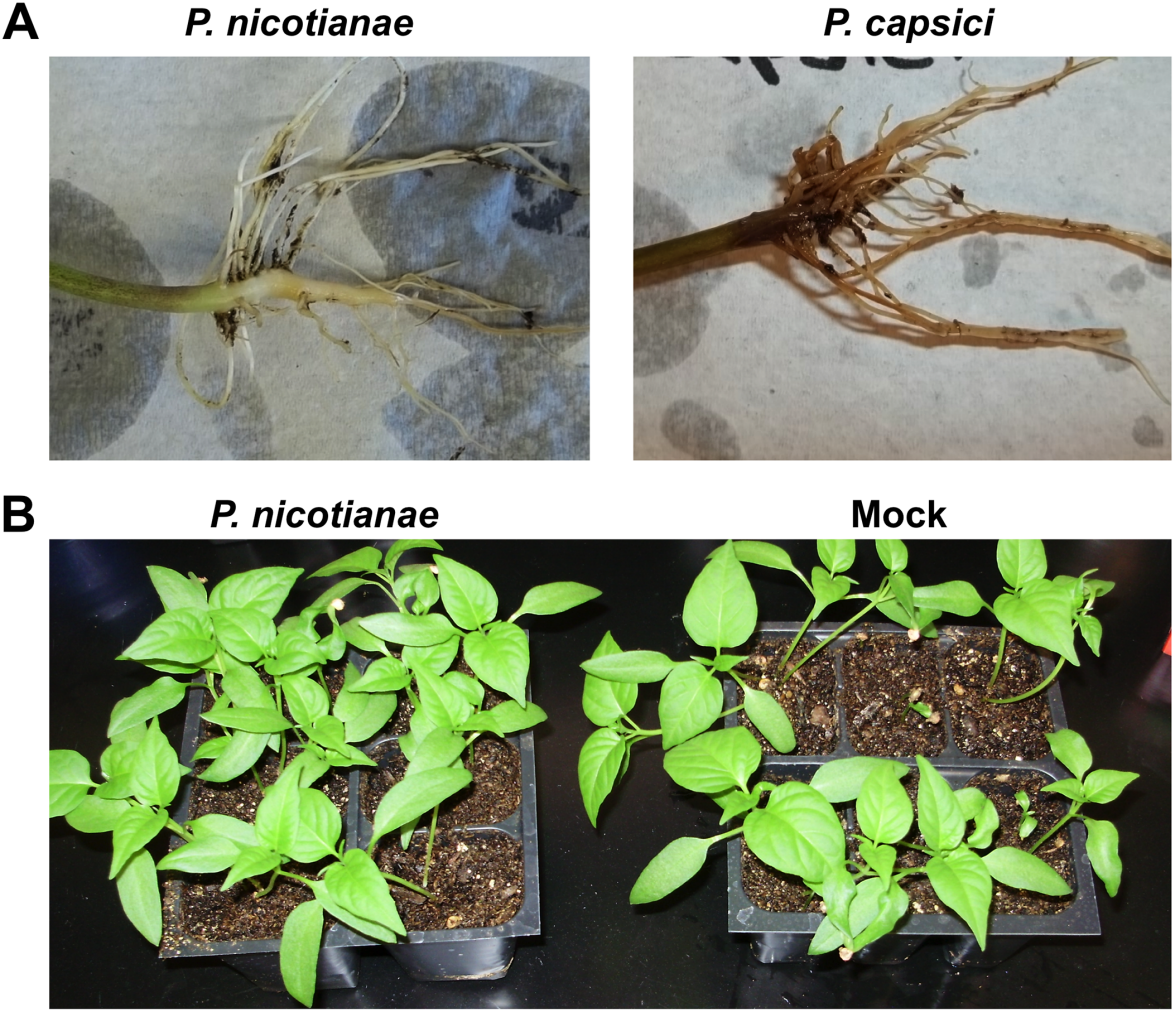
*Phytophthora nicotianae* pathogenicity assays on *Capsicum annuum* plants. (A) *Capsicum annuum* plants were soil inoculated with 375,000 *P*. *nicotianae* (isolate NMT1: Genbank: HQ711620) zoospores per plant or 10,000 *P*. *capsici* (strain PWB24: ATCC MYA-2289) zoospores per plant and watered daily for two weeks. (B) *Capsicum annuum* plants were foliar inoculated with 100,000 *P*. *nicotianae* zoospores or mock inoculated with water and incubated in a humidity chamber for 5 days at 28°C. No symptom development was observed on the crown, roots, or foliage of *P*. *nicotianae* inoculated plants.

Pathogenicity assays were conducted to determine if *P*. *nicotianae*, isolated from diseased tomato in New Mexico [12] was pathogenic on *C*. *annuum* cultivars that are susceptible to *P*. *capsici*. Roots of three *C*. *annuum* cultivars (Camelot, NM-64, and Jupiter) were inoculated by soil drenching each plant with a suspension of 375,000 *P*. *nicotianae* zoospores and plants were watered daily for two weeks. No disease symptoms were observed following inoculation with *P*. *nicotianae* (Fig 1). Similarly, no disease symptoms were observed following foliage spray with a suspension of 100,000 *P*. *nicotianae* zoospores per plant and incubation in a humidity chamber (Fig 1).

### Inoculation with non-host *P*. *nicotianae* or treatment with BABA elicits localized autofluorescent compounds and reduces photosynthetic rate in *C*. *annuum*

**Fig 2:**
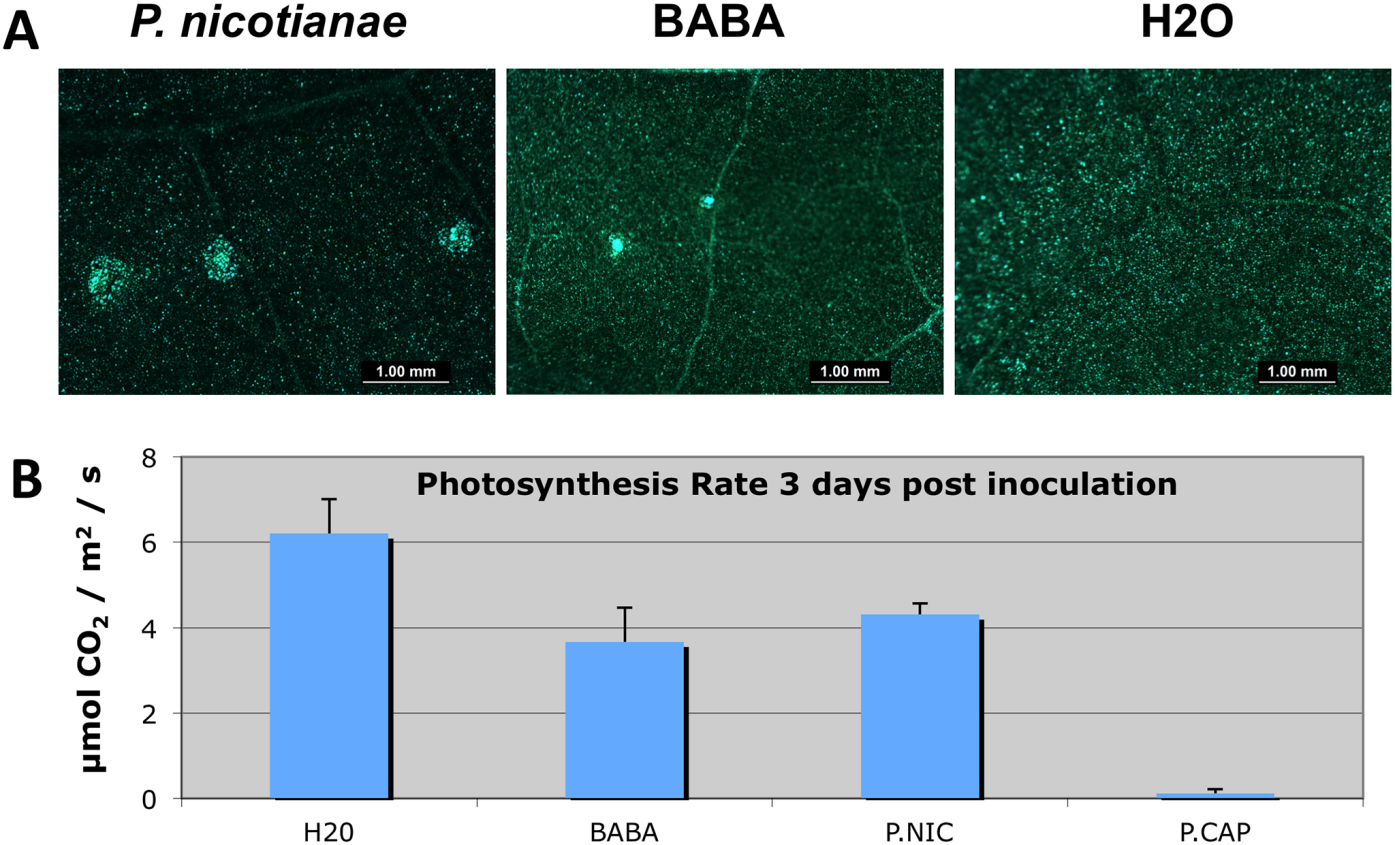
Treatment of *Capsicum annuum* with non-host *Phytophthora nicotianae* or BABA elicits localized autofluorescent compounds and reduces photosynthetic rate in *C*. *annuum*. (A) *C*. *annuum* leaves were inoculated with 2,000 *P*. *nicotianae* zoospores, 2.5 mM BABA or mock inoculated with water and incubated in a humidity chamber. After 48 hours, inoculated leaf tissue was excised and imaged using a stereofluorescent microscope. (B) *C*. *annuum* plants were soil drenched with *P*. *nicotianae* or *P*. *capsici* zoospore solutions (100,000 zoospores per plant), 2.5 mM BABA or water and photosynthetic rate was measured using a Licor 6400 portable photosynthesis system 3 days post inoculation. Standard deviation bars are shown.

*Capsicum annuum* leaves were sprayed with a suspension of 2,000 *P*. *nicotianae* zoospores, 2.5 mM BABA, or water (control) and incubated in a humidity chamber. After 48 h inoculated leaf tissue was excised and imaged using a stereo fluorescent microscope. Auto-fluorescent spots, less than 1 mM in diameter, were present on leaves inoculated with *P*. *nicotianae* or treated with BABA at the site of inoculation or treatment (Fig 2). No auto-fluorescence was visible outside of the inoculated or treatment area. Occasionally, small necrotic spots (< 1mm in diameter) developed at the site of inoculation with *P*. *nicotianae* or treatment with BABA (data not shown). *Capsicum annuum* plants were soil drenched with a zoospore suspension of *P*. *nicotianae* or *P*. *capsici* (100,000 zoospores per plant), 2.5 mM BABA or water, and photosynthetic rates were measured using a Licor 6400 portable photosynthesis system. At three days post inoculation or treatment, photosynthetic rates for plants treated with BABA and plants treated with *P*. *nicotianae* were approximately one third of the photosynthetic rate observed for the plants treated with water. In contrast, photosynthesis in plants inoculated with *P*. *capsici* was negligible (Fig 2).

### Soil drench with zoospore suspension of *P*. *nicotianae* or BABA inhibits *P*. *capsici* in *C*. *annuum*

**Fig 3:**
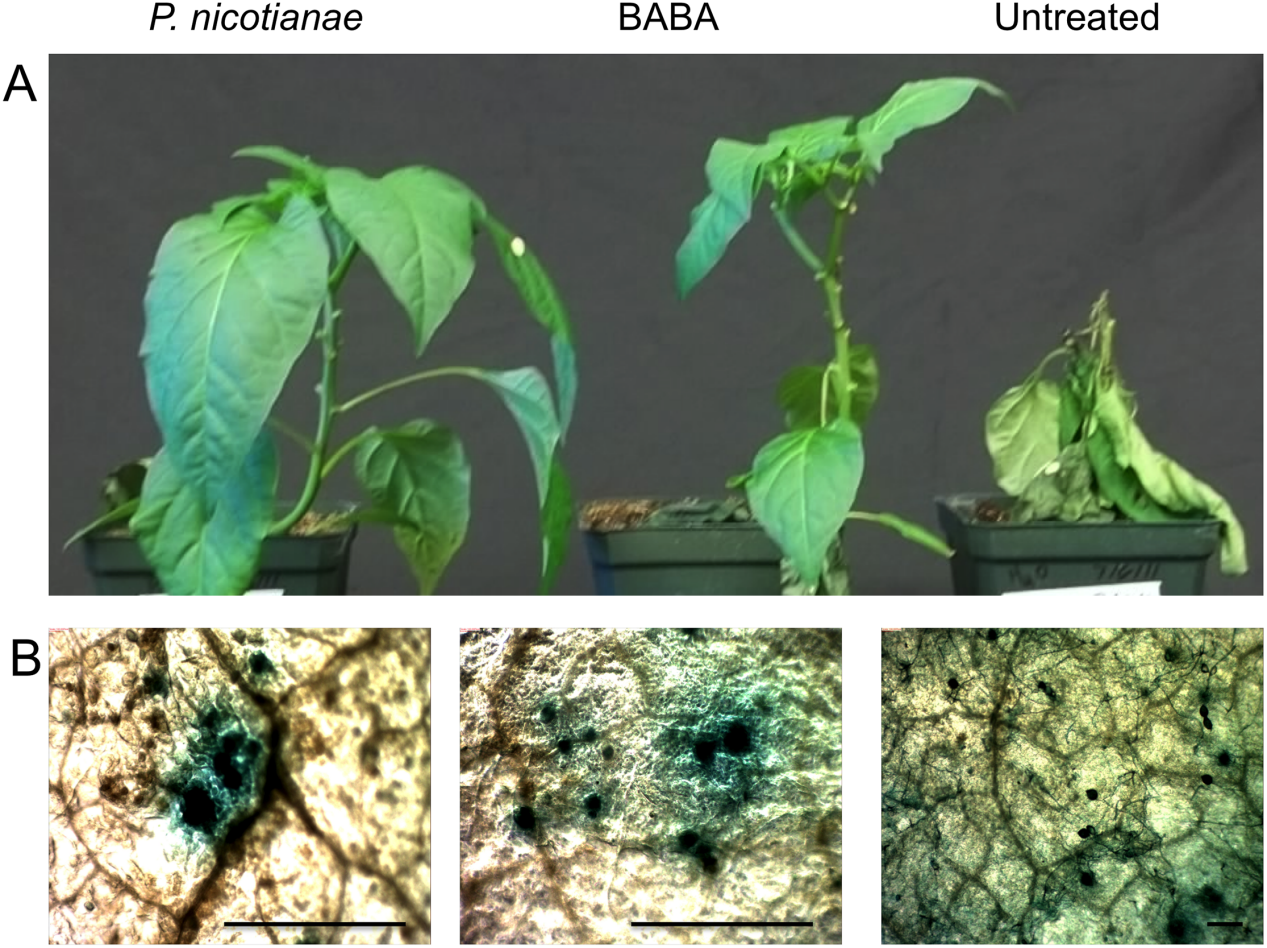
Differential response to *P*. *capsici* foliar inoculation with BABA or *Phytophthora nicotianae* treated *Capsicum annuum*. Plants were soil drenched with 100,000 *P*. *nicotianae* zoospores, 2.5 mM BABA, or water and foliar inoculated with 2,000 *P*. *capsici* zoospores per leaf, and plants were incubated in a humidity chamber at 28°C. for 48 h Plants were removed from humidity chamber and watered normally and grown under fluorescent light for an additional two weeks. (A) *P*. *capsici* caused systemic foliar blight and death in all untreated plants, whereas 50%- 83% of *P*. *nicotianae* or BABA treated plants did not develop disease symptoms outside of the inoculated leaves. (B) Histochemical x-gluc staining of GUS expressing *P*. *capsici* was utilized to visualize pathogen structures on inoculated *C*. *annuum* leaves. At 72 h post inoculation, abundant *P*. *capsici* hyphae and sporangia were visible on untreated plants, while BABA and *P*. *nicotianae* treated plants displayed confined areas of x-gluc staining with no identifiable *P*. *capsici* structures. Scale bar 30 μm for all images.

Three cultivars of *C*. *annuum*, (Camelot, NM-64, and Jupiter) were soil drenched with a suspension of 100,000 *P*. *nicotianae* zoospores, 2.5 mM BABA, or water, and subsequently foliar inoculated with a suspension of 2,000 zoospores of *P*. *capsici* per leaf. At 48 h post inoculation with *P*. *capsici*, all plants developed localized foliar symptoms consistent with a susceptible *P*. *capsici* / *C*. *annuum* interaction, with no visible differences among treatments (data not shown). At two weeks post inoculation with *P*. *capsici*, significant differences in disease progression were visible between treated and untreated plants (Fig 3). For the three *C*. *annuum* cultivars evaluated, *P*. *capsici* strain PWB 24 caused systemic foliar blight and death in all untreated plants, whereas 50%- 83% of plants treated with *P*. *nicotianae* or BABA did not develop disease symptoms outside of the inoculated leaves. Histochemical staining of GUS-expressing *P*. *capsici* was utilized to visualize pathogen structures on inoculated leaves. At 72 h post inoculation, abundant hyphae and sporangia of *P*. *capsici* were visible on untreated plants, while plants treated with BABA and *P*. *nicotianae* displayed confined areas of histochemical staining with no identifiable structures of *P*. *capsici* (Fig 3).

### Soil drench with zoospore suspension of *P*. *nicotianae* or BABA primes *C*. *annuum* foliar defense responses

**Fig 4:**
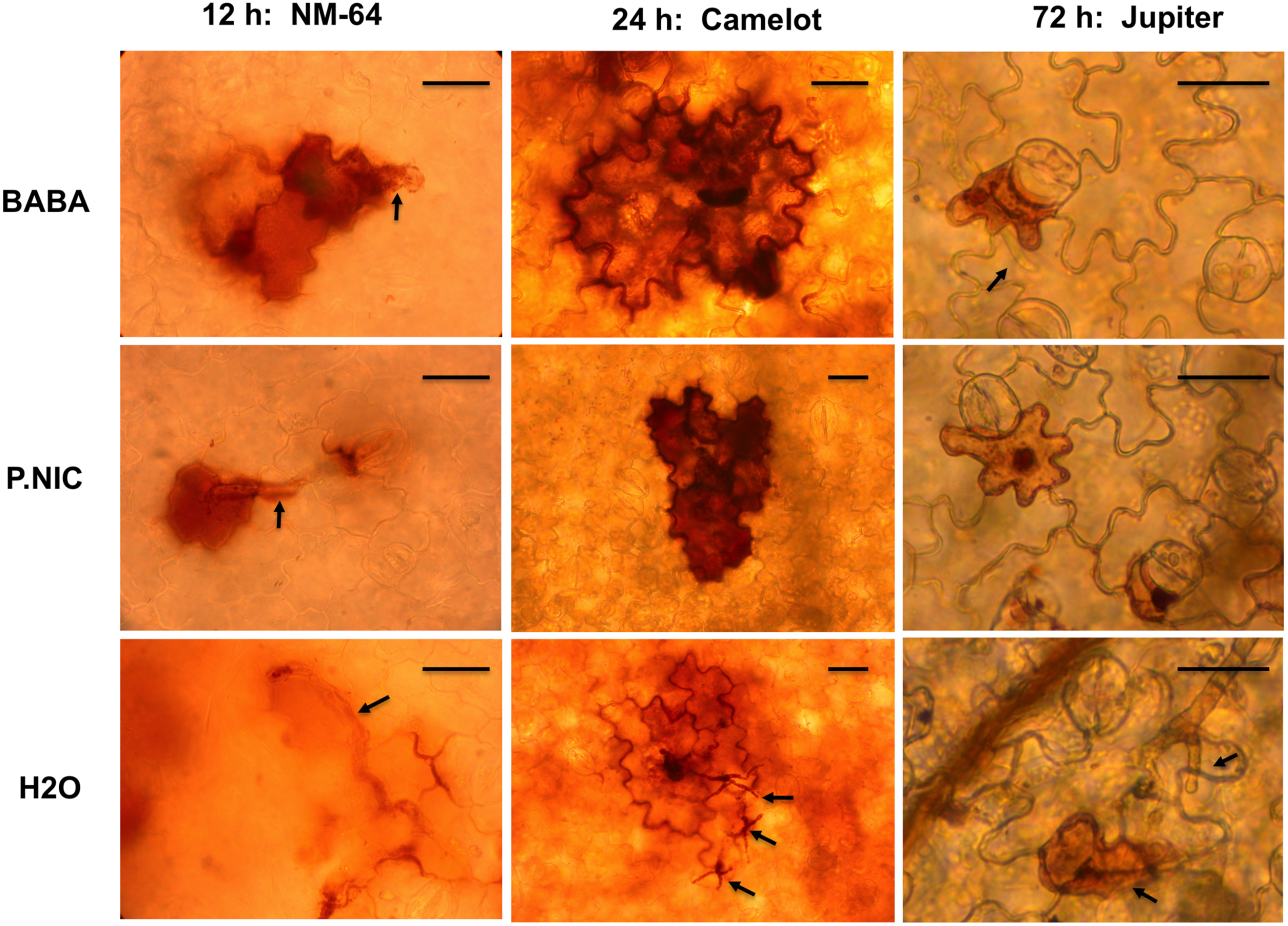
Enhanced production of hydrogen peroxide and development of hypersensitive response in BABA and *P*. *nicotianae* induced *C*. *annuum* plants during early *P*. *capsici* infection. The hydrogen peroxide indicator stain 3-3- diaminobenzidine (DAB) was used to visualize reactive oxygen species production at 12, 24, and 48 h post *P*. *capsici* inoculation. The presence of hydrogen peroxide is indicated by the formation of a dark precipitate. *P*. *capsici* hyphae and germtubes are indicated with arrows. Tissue was imaged using a compound microscope with a digital camera mounted using an eyepiece adapter (Nikon, Melville, NY). Scale bar is 10 μm for all images.

Production of autofluorescent compounds was evaluated in plants treated with *P*. *nicotianae* at 24 h post foliar inoculation with *P*. *capsici*. Confined autofluorescence in epidermal and mesophyll cells was observed, typically in close proximity to stomatal openings (data not shown). The hydrogen peroxide indicator stain 3-diaminobenzidine (DAB) was used to visualize reactive oxygen species production during initial *P*. *capsici* infection (Fig 4). At 12 h post inoculation with *P*. *capsici*, encysted zoospores with germination tubes were visible on all leaf surfaces. For plants treated with *P*. *nicotianae* and BABA, DAB staining was visible in single or multiple plant epidermal cells at or near the encysted zoospore and germ tube while staining in mock treated plants was indistinct, and often concentrated in the intercellular space between epidermal cells. At 24 h post inoculation, intense DAB staining was visible on induced plants within single or multiple epidermal cells near germinated zoospores, while DAB staining in mock treated plants remained indistinct and *P*. *capsici* hyphae could be seen emerging from leaf tissue. At 72 h post inoculation with *P*. *capsici*, induced plants had darkly stained punctate bodies within DAB stained epidermal cells. By 72 h post inoculation, mock treated plants also displayed DAB staining in epidermal cells, however, the punctate bodies were not present and abundant hyphae of *P*. *capsici* were present on the leaf surface. In addition, mock treated plants had a disorganized appearance and intercellular boundaries were not clearly defined. At all three time points, a limited number of infection sites were visualized in induced plants that were similar in appearance to mock treated plants and *P*. capsici hyphae were observed at low frequency (data not shown). However, no infection sites were visualized in mock treated plants that displayed similar DAB staining profiles as those shown in plants treated with BABA and *P*. *nicotianae*.

### Metabolic shifts associated with induced defense responses in *C*. *annuum*

**Fig 5:**
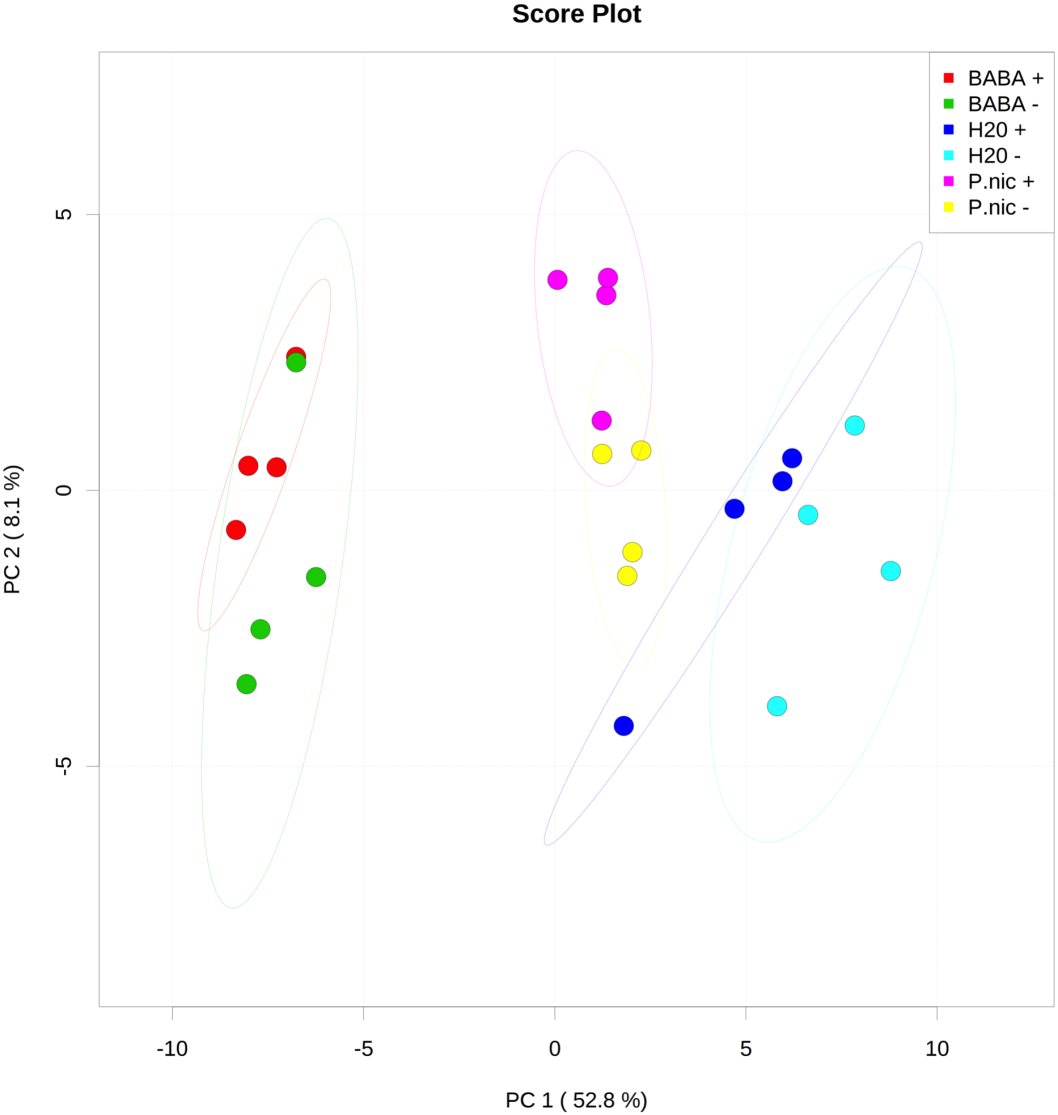
Principle components analysis (PCA) of relative concentrations for 62 metabolites identified in *Capsicum annuum* through GCMS analysis. *Capsicum annuum* seedlings were treated with BABA, *P*. *nicotianae* (P.nic), or water (H2O) and challenged with *P*. *capsici* (+) or mock inoculated with water (-) for a total of 6 treatments, with each treatment replicated on 4 blocks with 15 seedlings each in each block. Normalized peak area for each metabolite was log transformed and autoscaled to focus on relative changes across treatments. Substantial shifts in metabolite concentrations were identified for BABA treated plants, with no clear distinction between *P*. *capsici* (+) or mock challenged (-) groups. *P*. *nicotianae* (P.nic) induced plants that were challenged with *P*. *capsici* were distinguishable from non-induced (H2O) plants at the 95% confidence interval.

Changes in primary metabolite concentrations in *C*. *annuum* ‘Jupiter’ tissue extracts were identified by GC/MS analysis. In total, 62 compounds were identified and monitored for all samples. Principle component analysis (PCA) highlights dominant shifts in metabolite concentration for BABA treated plants (with no clear distinction between *P*. *capsici* challenged [BABA (+)] and mock challenged [BABA (-)] groups observed (Fig 5). At the 95% confidence interval, plants treated with *P*. *nicotianae* that were challenged with *P*. *capsici* [*P*.*nic* (+)] did not overlap with water treated plants [H20 (+/-)]. A post-hoc analysis of variance (ANOVA) with a p value of 0.05 identified 56 metabolite concentrations that were statistically distinct between BABA (+/-) and H20 (+/-), 26 metabolites were distinct between *P*. *nic* (+) and H20 (+/-) and six metabolites that were distinct between *P*.*nic* (+) and *P*.*nic* (-). Of the 26 metabolites that significantly changed in concentration for *P*.*nic* (+) compared to H20 (-), 25 were shifted in the same direction as BABA (+/-).

**Fig 6:**
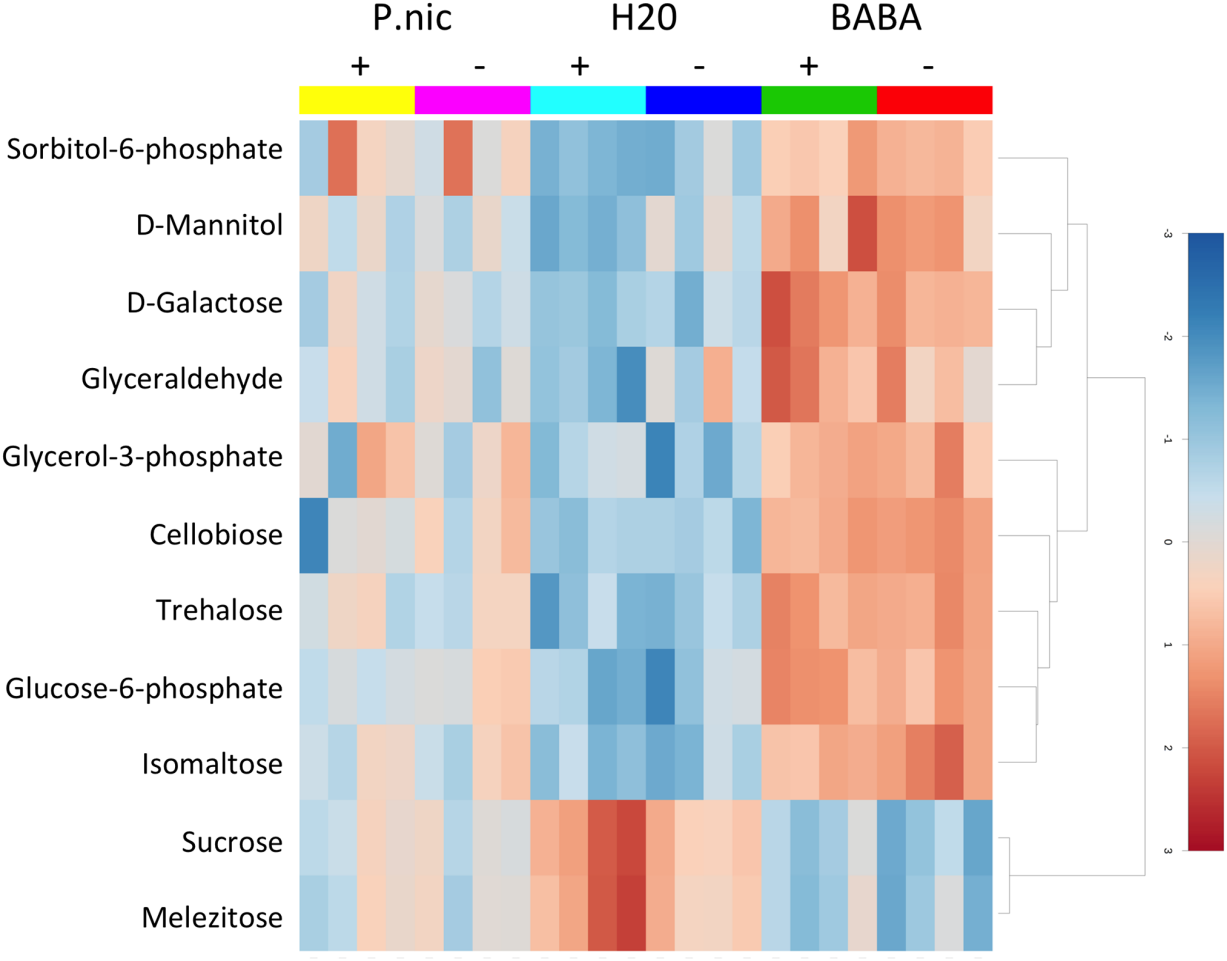
Heat map of carbohydrate metabolites that significantly changed in concentration for BABA and *P*. *nicotianae* induced plants. *Capsicum annuum* seedlings were treated with BABA, *P*. *nicotianae* (P.nic), or water (H2O) and challenged with *P*. *capsici* (+) or mock inoculated with water (-) for a total of 6 treatments, with each treatment replicated on 4 blocks with 15 seedlings each in each block. Metabolites in red indicate higher relative concentrations while those in blue indicate lower relative concentrations.

**Fig 7:**
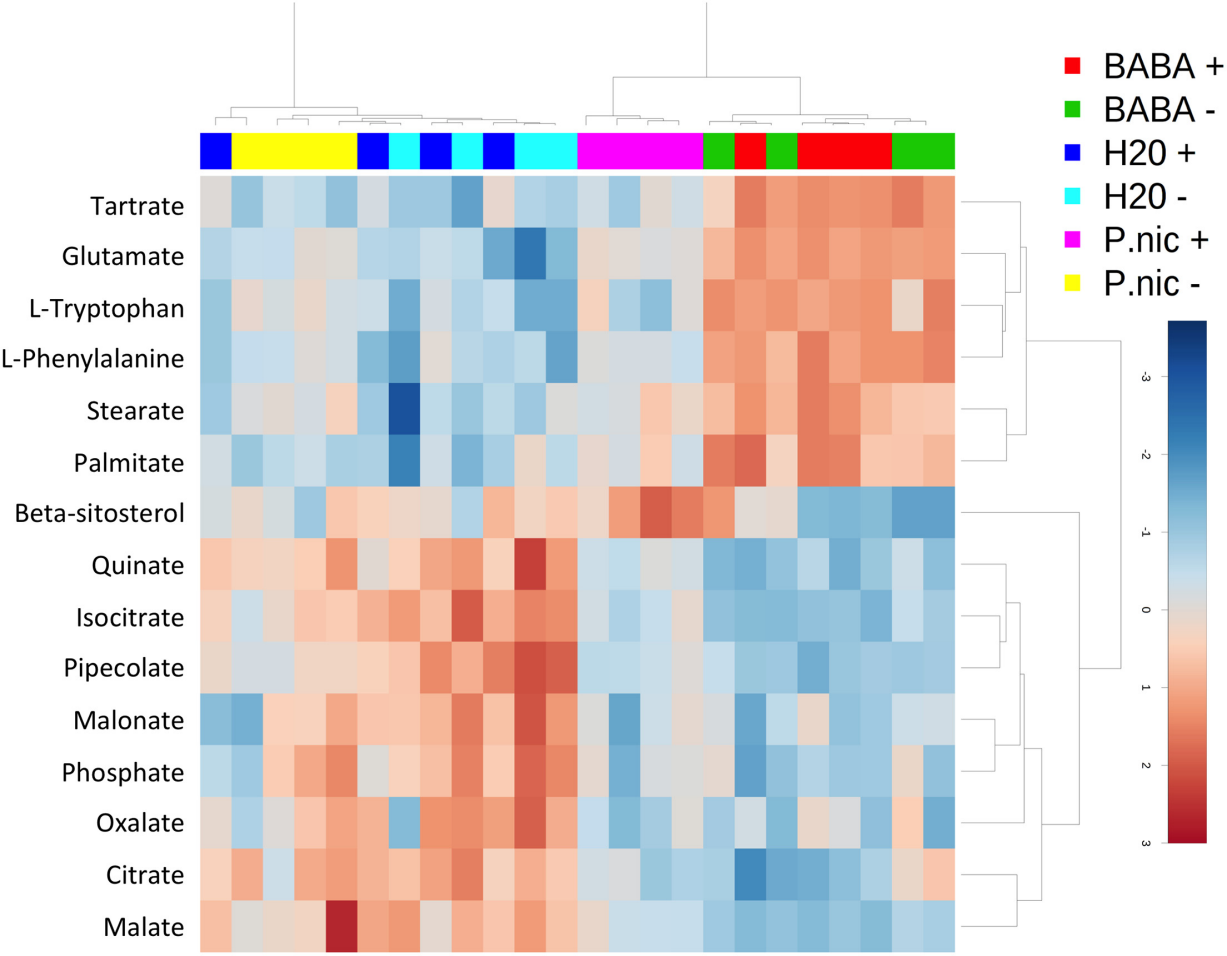
Heat map and dendrogram of non-carbohydrate metabolites that significantly changed in *Phytophthora nicotianae* induced *Capsicum annuum* plants. *Capsicum annuum* seedlings were treated with BABA, *P*. *nicotianae* (P.nic), or water (H2O) and challenged with *P*. *capsici* (+) or mock inoculated with water (-) for a total of 6 treatments, with each treatment replicated on 4 blocks with 15 seedlings each in each block. Dendrogram was constructed using the Spearman distance measure and Ward clustering algorithm. Metabolites in red indicate higher relative concentrations while those in blue indicate lower relative concentrations.

**Fig 8:**
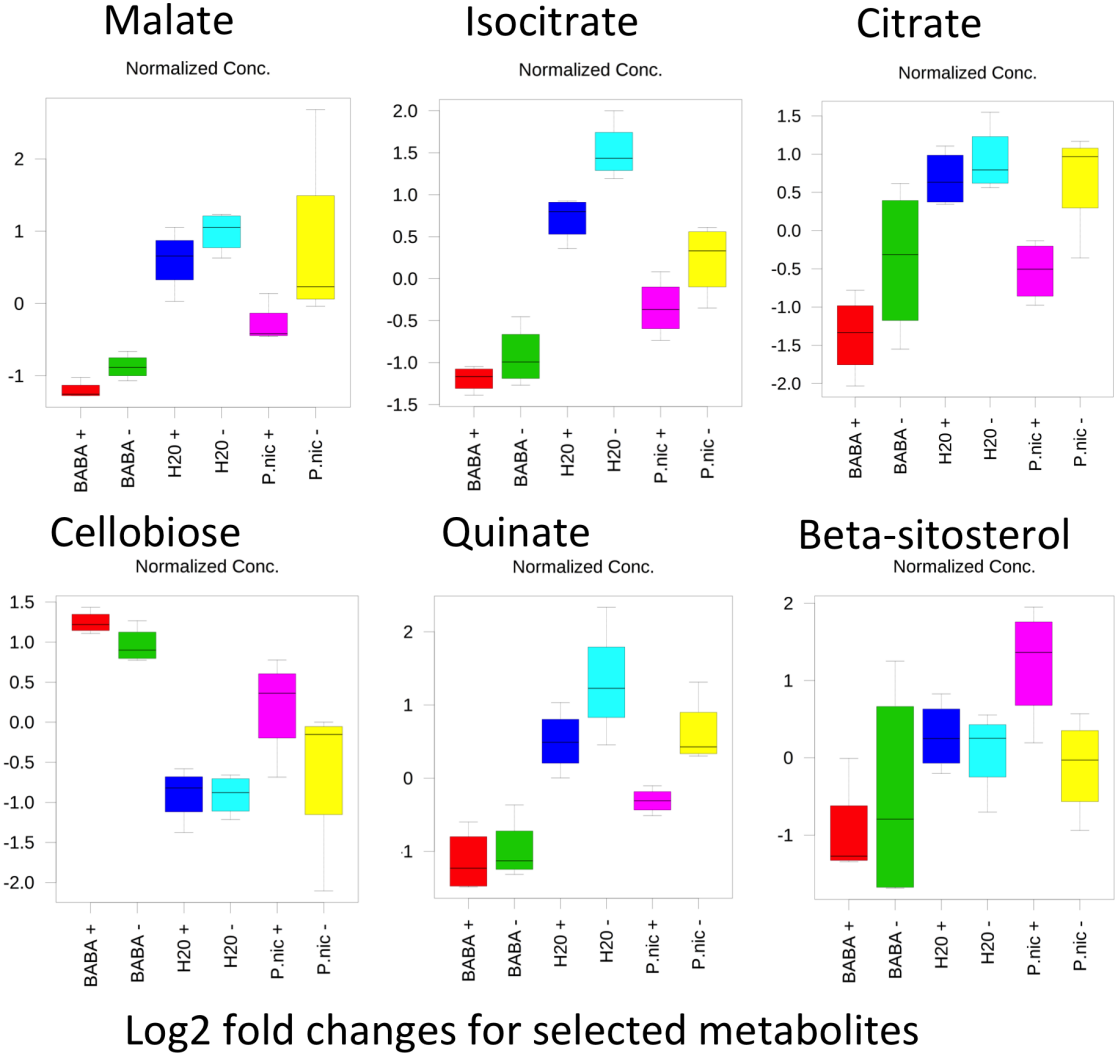
Box and whisker plots for metabolites that were statistically distinct between *Phytophthora nicotianae* induced plants that were challenged with *P*. *capsici* [*P*.*nic* (+)] and mock inoculated plants [*P*.*nic* (-)]. Data were log transformed and autoscaled (mean centered and divided by the standard deviation of each variable) to focus on relative changes in metabolite profiles across treatments.

Twelve of the metabolites that were significantly shifted in *P*. *nic* (+/-) treated plants compared to H20 (+/-) plants were carbohydrates. Induced plants had reduced concentrations of sucrose and melezitose and increased concentration of sorbitol-6-phosphate, mannitol, galactose, glyceraldehyde, glycerol-3-phosphate, cellobiose, trehalose, glucose-6-phosphate and isomaltose (Fig 6). There were no significant differences in carbohydrate concentrations between H20 (+) and H20 (-) treatments. The remaining 14 compounds that were significantly shifted between *P*.*nic*(+) and H20(-) include aromatic amino acids, lipids, TCA-cycle intermediates and phosphoric acid. Dendrogram analysis of non-carbohydrate metabolites grouped *P*. *nic*(+) treated plants with BABA(+/-) treated plants, while *P*.*nic*(-) treated plants were grouped with H20 (+/-) treated plants (Fig 7). In total, six metabolites were statistically distinct between *P*.*nic* (+) and *P*.*nic* (-) treated plants (Fig 8). The TCA-cycle intermediates, isocitrate, citrate and malate significantly decreased in concentration for plants treated with *P*. *nicotianae* and challenged with *P*. *capsici*. In addition, cellobiose, and beta-sitosterol increased in concentration while quinate decreased in concentration for *P*.*nic*(+) plants compared to *P*.*nic*(-) plants. Metabolite concentrations that were shifted in both *P*.*nic*(+) and *P*.*nic*(-) plants included increases in tartrate, glutamate, tryptophan, phenylalanine, octadecanoate and hexadecanoate and decreased concentrations of pipecolate, malonate, and oxalate. Table 1 lists all metabolites with fold changes greater than 2 between BABA and H20 treated plants.

**Table 1:** Metabolites with fold changes greater than 2 between BABA and water treated *C*. *annuum* plants.

Metabolite Name	Signature Ion	Elution time	Fold Change
Malonic Acid	234	9.5	0.4
L-Alanine	190	9.7	2.9
L-Valine	604	9.9	2.3
Ethanol Amine	174	11.2	2.5
Phosphoric Acid	299	11.3	0.4
L-Isoleucine	158	11.9	2.5
Succinic Acid	247	12.4	4.4
Propionic Acid	360	12.7	0.5
L-Serine	204	13.8	32.1
Allothreonine	218	14.5	6.8
Pipecolic Acid	175	17.0	0.1
Malic Acid	246	17.2	0.2
Pyroglutamic Acid	260	17.5	3.3
L-Aspartic Acid	232	17.7	9.8
4-Aminobutyric Acid	174	17.7	3.4
L-Phenylalanine	218	19.8	14.5
L-Glutamic Acid	246	20.0	31.0
Citric Acid	217	24.6	0.4
Isocitric Acid	319	24.6	0.3
Xylose	308	25.8	2.2
D-Psicose	219	25.8	2.1
D-Glucose	205	26.1	7.2
Ascorbic Acid	157	26.1	2.8
Glyceraldehyde	319	26.3	3.0
D-Mannose	205	26.6	2.7
Hexadecanoic Acid	313	28.2	5.7
D-Galactose	319	30.7	6.2
L-Tryptophan	202	31.1	158.8
n-Octyl-beta-D-glucoside	205	31.7	2.0
Sorbitol-6-Phosphate	204	33.7	3.6
Glucose-6-Phosphate	299	33.9	9.2
Cellobiose	204	35.9	3.0
ISOMALTOSE	290	37.2	3.0
Sucrose	169	39.0	0.4
Xylulose	161	39.3	2.0
Trehalose	271	40.3	3.9
1-Octadecane	327	44.1	5.0
Laminaribose	363	46.2	65.0

### Galactose significantly inhibits growth of *P*. *capsici* in-vitro

**Fig 9:**
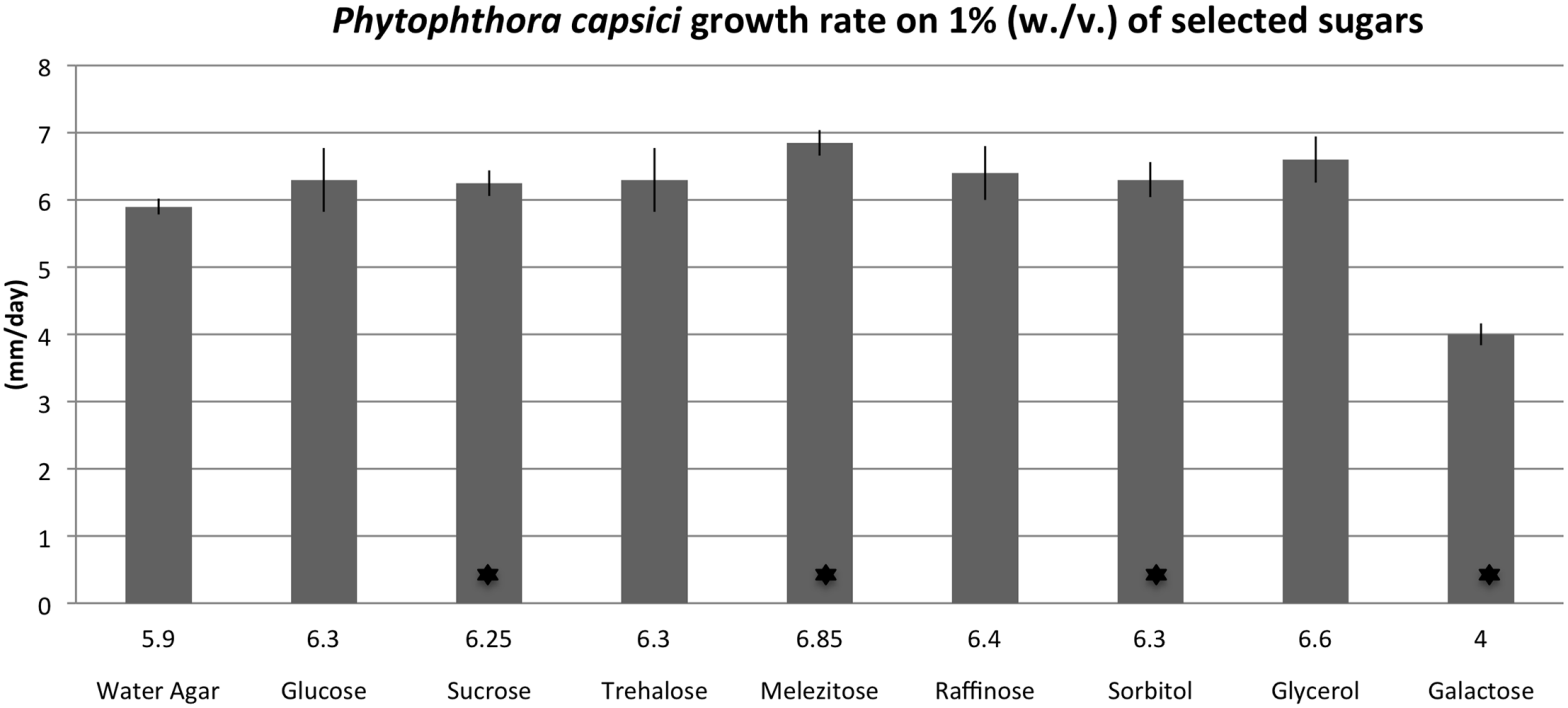
Impact of selected carbohydrates on *Phytophthora capsici* growth rate. *P*. *capsici* colonies were grown on water agar supplemented with 1% (w / v) of listed sugars at 28°C for five days. Average growth (mm / day) rate is listed above each label. * Indicates significant differences in growth rate compared to water agar, α equal to 0.05.

To evaluate the impact of various sugar sources on growth rate, *P*. *capsici* was grown on water agar supplemented with 1% (w / v) of selected sugars. Growth rate of *P*. *capsici* was approximately 30% slower on 1% galactose compared to water agar (Fig 9). There was a small but significant increase in *P*. *capsici* growth rate on 1% sucrose, melezitose, and sorbitol.

## Discussion

This study represents the first evaluation of non-host *P*. *nicotianae* as an elicitor of induced resistance in *C*. *annuum*. Consistent with previously evaluated chemical and biological elicitors, treatment with BABA and *P*. *nicotianae* inhibited subsequent foliar infection by *P*. *capsici* in a cultivar independent manner. No macroscopic differences were observed between treated and non-treated plants during initial foliar symptom development. However, systemic progression of *P*. *capsici* was halted in more than 50% of treated plants while all untreated plants developed systemic foliar blight resulting in plant death. As the virulent gene-for-gene interaction is unchanged by elicitor treatment, the difference in virulence of *P*. *capsici* can be presumed to be associated with enhanced basal defense responses including production of oxidative compounds and initiation of a hypersensitive response.

Metabolite analysis by GC/MS identified significant shifts in metabolite concentrations in plants treated with BABA and *P*. *nicotianae*. Changes in metabolite concentrations were more pronounced for plants treated with BABA than for plants treated with *P*. *nicotianae*, both in magnitude and in the number of metabolites that changed significantly. Changes in metabolite concentrations induced by BABA is consistent with previous studies in other plant systems where pronounced differences were measured within 24 hours after treatment [8]. Consistent with previous reports, we observe parallel concentration responses for 25 metabolites (of 26 total for which significant concentration change was observed) in plants treated with *P*. *nicotianae* and BABA, which indicates a conserved response mechanism for both elicitors [13]. Primed changes were observed in plants treated with *P*. *nicotianae* that were subsequently inoculated with *P*. *capsici*. As the intracellular localization of metabolites cannot be determined through this analysis, it is difficult to determine their exact biochemical origin within the plant. However, the observed metabolite changes are consistent with primed production of defense compounds and to supply the necessary reducing power to generate reactive oxygen species involved in defense response. In addition, changes in carbohydrate concentrations were previously associated with signal transduction pathways responsible for enhanced immune response to pathogen infection [14].

**Fig 10:**
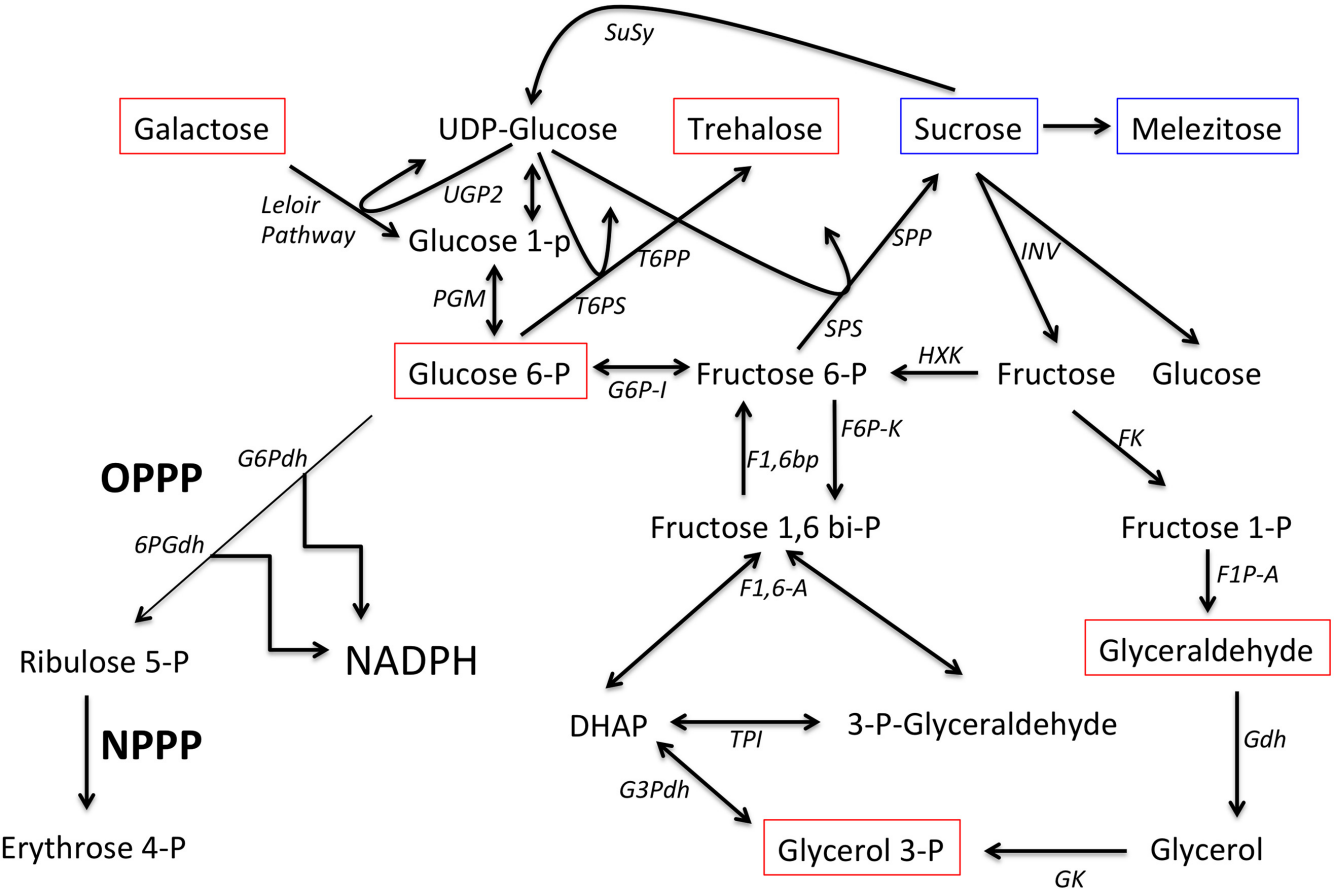
Overview of selected carbohydrate pathways. Metabolites boxed in red were significantly increased in concentration in induced plants while metabolites boxed in blue were significantly decreased. Abbreviations: oxidative pentose phosphate pathway (OPPP), non-oxidative pentose phosphate pathway (NPPP), sucrose synthetase (SuSY), UTP glucose-1-phosphate uridylyltransferase (UGP2), phosphoglucomutase (PGM), trehalose 6-phosphate synthase (T6PS), Trehalose 6- phosphate phosphatase (T6PP), sucrose phosphate synthase (SPS), sucrose phosphate phosphatase (SPP), invertase (INV), glucose 6-phosphate isomerase (G6P-I), hexose kinase (HXK), fructose kinase (FK), fructose 1-phosphate aldolase (F1P-A), glyceraldehyde dehydrogenase (Gdh), fructose 6-phosphate kinase (F6P- K), fructose 1,6 bisphosphatase (F1,6bp), fructose 1,6 bisphosphate aldolase (F1,6- A), triose phosphate isomerase (TPI), glycerol 3-phosphate dehydrogenase (G3Pdh), glycerol kinase (GK), glucose 6-phosphate dehydrogenase (G6Pdh), 6- phosphogluconate dehydrogenase (6PGdh).

Carbohydrate pools likely provide the carbon source for the production of aromatic amino acids and oxidative compounds via increased flux through the pentose phosphate pathway (PPP) (Fig 10). Glucose-6-phosphate, which increased in induced plants in this study, is the primary precursor for the PPP. The oxidative portion of the PPP is a significant source of NADPH that provides reducing power for the generation of reactive oxygen species through NADPH oxidases. The non-oxidative portion of the PPP generates erythrose 4-phosphate which is the primary precursor to the shikimate pathway for aromatic amino acids. The increased pools of tryptophan and phenylalanine are precursors for defense related antimicrobial and plant signaling compounds such as lignins, alkaloids, chalcones, salicylic acid, and auxins.

**Fig 11:**
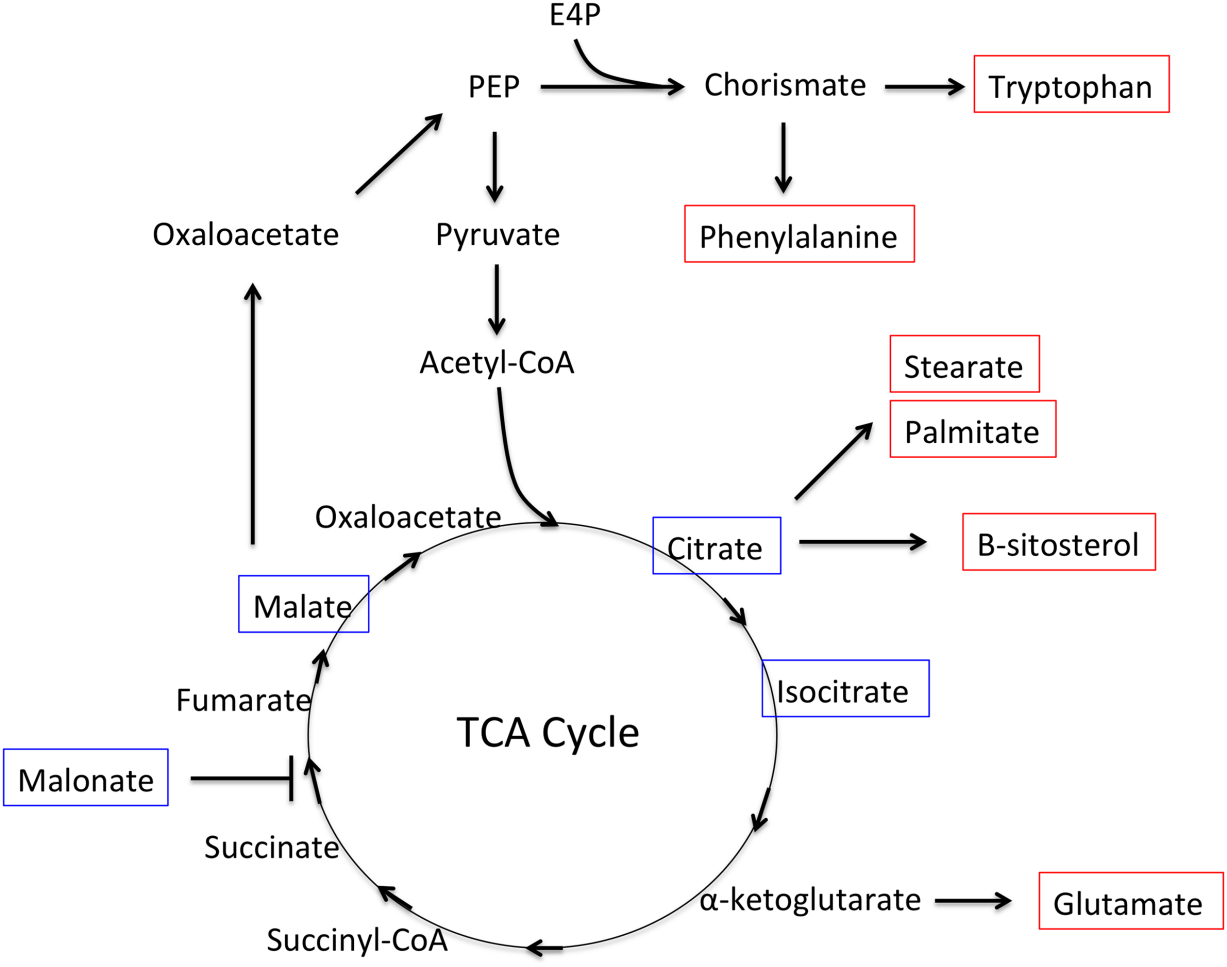
Overview of TCA cycle and associated biochemical pathways. Metabolites boxed in red were significantly increased in concentration in induced plants while metabolites boxed in blue were significantly decreased.

Increased invertase activity, which cleaves sucrose into glucose and fructose, has been reported following pathogen perception in plants [14–15]. This may explain the decrease in sucrose concentration (approximately 1.5-fold decrease) observed in induced plants in this study. However, given that sucrose is the primary transport sugar in plants, it is difficult to interpret changes in concentration without additional labeling studies. Laminaribose, which increased significantly in BABA treated plants, was previously shown to inhibit cell death and browning in potato tubers inoculated with *P*. *infestans* ([16]. Increased concentration of the small metabolite glycerol-3-phosphate, is consistent with recent reports that it acts as a signaling molecule during induced resistance in *A*. *thaliana* [17–18]. Increased trehalose concentrations observed in induced plants is also consistent with reports that it is involved in stress response signaling and leads to enhanced osmotic protection [19]. Trehalose 6-phosphate, a precursor to trehalose, appears to be an important signaling molecule for embryo development, plant growth and senescence processes, and is linked to alterations in carbohydrate metabolism, amino acid, protein, nucleotide synthesis, the tricarboxylic acid (TCA) cycle and mitochondrial electron transport chain [20–22]. The TCA-cycle intermediates, citrate, isocitrate and malate were all significantly reduced in induced plants as was the TCA cycle inhibitor malonate. In addition, glutamate, stearate, palmitate and β-sitosterol all increased in concentration in induced plants. Together, these observations suggest increased flux through the TCA cycle, with intermediates being drawn off for synthesis of lipids and glutamate (Fig 11).

Significant change observed in carbohydrate levels with an induced defense response prompted a further study to determine if the presence of these carbohydrates would affect growth of *P*. *capsici*. To our knowledge, these data provide the first evidence that the carbohydrate galactose can directly inhibit growth of *P*. *capsici*. While it is unknown if the increased concentration of galactose observed in induced plants is sufficient to inhibit growth of *P*. *capsici* after infection, several reports indicate that galactose metabolism may play an important role in infection by *Phytophthora* spp. A late blight resistant hybrid potato line (*Solanum phureja* X *Solanum tuberosum*) was shown to differentially overexpress a gene encoding for α-galactosidase upon infection by *P*. *infestans* [23]. The α-galactosidase gene, also known as melibiase, cleaves the polysacharide melibiose to produce galactose and glucose. While the role of α-galactosidase in plant resistance is unknown, its over-expression during infection by *P*. *infestans* indicates that sugar metabolism is involved in plant-pathogen interactions. In *P*. *sojae*, a putative UDP-glucose 4-epimerase open reading frame (ORF) was identified in close proximity to a necrosis inducing protein and was shown to be actively expressed in zoospores and during pathogenesis [24]. Interconversion of UDP-galactose to UDP-glucose by UDP-glucose 4-epimerase is an essential step during galactose catabolism and its expression during early infection indicates that limiting galactose accumulation in *Phytophthora* spp. may be important for pathogenicity. In *Saccharomyces cerevisiae*, mutant lines with increased uptake of galactose accumulated galactitol and had a lower maximum growth rate compared to wild type cells [25]. Our results indicate that galactose is an inhibitor of *P*. *capsici* under *in-vitro* growth conditions, and could enable novel strategies for *Phytophthora* disease control.

## Materials and Methods

### *Phytophthora* spp. isolates and growth

*Phytophthora capsici* strain PWB24 (ATCC MYA-2289) and *P*. *nicotianae* isolate NM.T1 (Genbank: HQ711620) were used in this study. Isolates were maintained on clarified V8 agar, amended with Pimaricin (0.2% w./v.), Rifampicin (10 mg/L), and Ampicillin (250 mg/L) (cV8S) at 25°C. For sporangia production, 10 one-cm plugs were transferred from five-day-old colonies to 100 X 20 mm petri plates with 25 mL sterile DI water and incubated under fluorescent light at 25°C for 24 h. Zoospores were induced by incubating plates at 4°C for thirty min and at room temperature for 2–4 hours. Zoospore solutions were filtered through cheesecloth and quantified using a haemocytometer (Hausser Scientific, Horsham, PA).

### Pathogenicity Assays

Three *C*. *annuum* cultivars (Camelot, NM-64, and Jupiter) were soil drenched with a suspension of 375,000 zoospores of *P*. *nicotianae* per plant or a suspension of 10,000 zoospores of *P*. *capsici* per plant, and watered daily for two weeks. For foliar inoculation, 100,000 zoospores of *P*. *nicotianae* zoospores or mock inoculum (water) were sprayed onto *C*. *annuum* foliage and incubated in a humidity chamber at 28°C for 5 days. Eight plants per cultivar were used in each treatment group and the assay was replicated at an independent time point.

### Induced Resistance Assays

*Capsicum annuum* cultivars NM-64, Camelot, and Jupiter were grown in 10 cm pots with metro mix 360 potting soil (Sun Gro Horticulture Canada Ltd, Agawam, MA) under cool-white fluorescent light with a 18 and 6 h light and dark schedule at 28°C and fertilized with Osmicote at the recommended rate (The Scotts Company LLC, Marysville OH). Eight plants from each cultivar were inoculated at 4–6 true leaves for the split-plant assays utilized in the experiments described and the assay was replicated at a separate time point for all cultivars. Plants were soil drenched with 100 mL of a zoospore suspension of *P*. *nicotianae* (2,000 zoospores / mL), 2.5 mM BABA, or sterile DI water, and placed in trays with two cm standing water to maintain saturated soil conditions. Forty-eight hours after soil drench, plants were foliar inoculated on three separate leaves with 50 μL of a zoospore suspension of *P*. *capsici* (40,000 zoospores / mL) and incubated in a humidity chamber at 28°C for 48 h [26].

### Autofluorescence Evaluation

Autofluorescence in leaf tissue was evaluated 48 h after foliar treatment with *P*. *nicotianae* or BABA. Leaf discs were removed from the plant and imaged using an M165 FC Stereofluorescence microscope with an integrated CCD color camera system. Autofluoresence was also evaluated in plants that were soil drenched with a zoospore suspension of *P*. *nicotianae* or BABA, and foliar inoculated with *P*. *capsici* as described above. At 24 h post inoculation, leaf tissue was harvested and imaged directly using a compound fluorescent microscope.

### Photosynthesis Measurements

*Capsicum annuum* plants were soil drenched with a zoospore suspension of *P*. *nicotianae* or *P*. *capsici* (100,000 zoospores per plant), 2.5 mM BABA or water (six plants per treatment) and photosynthetic rate (μmol CO_2_ / m^2^ / s) was measured using a LI 6400 portable photosynthesis system according to manufacture’s instructions (LI-COR Inc. Lincold, NE). At three days post inoculation, three fully expanded leaves from each plant were analyzed using the sensor head with attached light and data were graphed using Microsoft Excel for Mac 2011 with standard deviation shown.

### Diaminobenzidine (DAB) staining

Plants were soil treated with a zoospore suspension of *P*. *nicotianae*, 2.5 mM BABA, or sterile DI water, and foliar inoculated with *P*. *capsici* a zoospore suspension as described above. At 12, 24, and 48 h post inoculation, leaf tissue was removed and immediately immersed in DAB solution (1 mg/mL) in 10 mM sodium phosphate buffer with Tween 20 (0.05% v/v) in a covered dish on a rocker platform at room temperature. After 4 h the DAB staining solution was replaced with bleaching solution (ethanol:acetic acid:glycerol 3:1:1) and boiled for 10 min to remove plant pigments [27]. Tissue was imaged using a compound microscope with a digital camera mounted using an eyepiece adapter (Nikon, Melville, NY).

### Metabolite analysis of *C*. *annuum* tissue

*Capsicum annuum* (*var*. Jupiter) seedlings with one true set of leaves were treated with *P*. *nicotianae*, BABA, or water (mock treatment), and after 48 h they were challenged with *P*. *capsici* (+) or mock inoculated with water (-) for a total of 6 treatments. Leaf tissue was harvested from plants in all treatments at 96 h following initiation of each treatment. A random block design was implemented with four blocks per treatment, each composed of 15 seedlings growing in sterile vermiculite under cool white fluorescent light at 28°C. Leaf tissue for the 15 seedlings in each block was harvested into a single container and immediately flash frozen in liquid nitrogen. Primary metabolites were extracted and analyzed by GC/MS according to previously described protocol with ~6.0 mg of lyophilized plant tissue [28]. All of the samples were analyzed using a Hewlett Packard 5890 GC with a 5972A mass selective detector. Polar samples were injected at a 15:1 split ratio and non-polar samples were injected at a 1:1 split ratio; the inlet and transfer line were held at 280°C. Separation occurred with an oven temperature program of 80°C held for 2 min then ramped 5°C per min to 315°C and held for 12 min for a total run time of 64 min. A 30 meter RTX-5 column (Restek, 0.25 mm ID, 0.25um film thickness) was used with a constant flow of 1.0 mL per minute. All GC data files were grouped by polarity and exported from the Automated Mass Spectral Deconvolution and Identification System (AMDIS). Next, polar and non-polar component data were aligned with Metabolomics Ion-based Data Extraction Algorithm (MET-IDEA). Component signals were aligned across all samples for comparative analysis and abundance data were normalized to tissue weight and relative concentration of spiked internal standards. Mass spectral matching was performed with the Samuel Roberts Noble Foundation metabolite library [28]. MetaboAnalyst 2.0 was used to for multivariate and univariate statistical analyses [29]. Data were log transformed then mean-centered and divided by the standard deviation of each variable to highlight relative changes in metabolite profiles across treatments. Data were analyzed by a Fisher’s LSD post-hoc analysis of variance (ANOVA) with α equal to 0.05 and a principle components analysis with 95% confidence threshold. Dendrograms were constructed using the Spearman distance measure and Ward clustering algorithm.

### Evaluation of selected carbohydrates on growth rate of *P*. *capsici*

Carbohydrate plates were prepared by autoclaving 12 g agar per L and 1% (w.v.) glucose, sucrose, melezitose, galactose, trehalose, raffinose, sorbitol, or glycerol with 10 mL of media poured in each plate. A single two mm plug from the edge of a *P*. *capsici* colony growing on water agar, was transferred to each plate with four replicates of each sugar. Plates were sealed with parafilm and incubated at 28°C for 5 days. The experiment was repeated with independently prepared plates at a separate time point and two measurements were taken for the diameter of each colony. Data were analyzed by a student’s t test with α equal to 0.05 to determine significant differences in growth rate on selected sugars compared to water agar and graphed using Microsoft Excel for Mac 2011.

## Supporting Information

S1 Text Data table containing metabolite concentrations used for Figs 5, 6, 7 and 8. Data were normalized to tissue weight and relative concentration of spiked internal standards and were then log transformed, mean-centered and divided by the standard deviation of each variable to highlight relative changes in metabolite profiles across treatments.
(XLSX)
